# Growth performance of five different strains of Nile tilapia (*Oreochromis niloticus*) introduced to Tanzania reared in fresh and brackish waters

**DOI:** 10.1038/s41598-021-90505-y

**Published:** 2021-05-27

**Authors:** Mbiru Moses, Leonard J. Chauka, Dirk Jan de Koning, Christos Palaiokostas, Matern S. P. Mtolera

**Affiliations:** 1 Institute of Marine Sciences, University of Dar es Salaam, P.O Box 668, Zanzibar, Tanzania; 2 Department of Aquaculture Development, Ministry of Livestock and Fisheries, P.O Box 670, 40404 Dodoma, Tanzania; 3 Department of Animal Breeding and Genetics, Swedish University of Agricultural Sciences, 750 07 Uppsala, Sweden

**Keywords:** Marine biology, Ecosystem ecology

## Abstract

Five introduced strains of Nile tilapia (*Oreochromis niloticus*) were tested for growth performance both in fresh- and brackish-water (2 salinity units) environments for 56 days. The BIG NIN, GIFT, Chitralada, “Ruvu Farm” and Silver YY strains with initial mean average weight (± standard error) of 96.4 ± 6.90 g, 104.1 ± 7.19 g, 137.2 ± 7.21 g, 53.2 ± 6.98 g and 95.3 ± 7.11 g, respectively were used. Individuals were tagged and pooled in hapas (12 m × 8.5 m × 2 m each), aligned into different ponds (20 m × 20 m each). Stocking density of 5 fish/m^2^ and 350 g/kg crude protein diet were used. Overall, the average weight gain for GIFT strain was 7.5%, 32%, 45% and 86.5% higher than BIG NIN, Chitralada, “Ruvu Farm” and Silver YY strains, respectively, across both environments. All strains performed significantly better (p < 0.05) when reared in brackish-water than their respective counterparts in freshwater, except for the BIG NIN strain. The morphometric correlations for all strains in both environments ranged from moderate (0.50) to strong positive (0.92). The GIFT strain demonstrated superior growth and genotype by environment interaction was weak and not important to be prioritized in breeding programs.

## Introduction

Tilapias, especially Nile tilapia (*Oreochromis niloticus*)*,* have the potential to become the leading farmed fish species in the world^1^. The species has been constantly expanding in the last three decades and presently is reported in 87 countries around the world, with an estimated production of 4.2 million tonnes in 2016^2^. The production bulk originates from diverse aquaculture systems, although earthen ponds remain the most commonly used rearing means for tilapia aquaculture. Such other aquaculture systems include; above ground and raceway tanks, cages and recirculating aquaculture system (RAS). Evolution of its aquaculture research in the 1970s to mid-1980s started by targeting seed production technology and improvement of fish husbandry rather than genetic improvement^3^. Consequently, some farmed strains of Nile tilapia suffered inbreeding and introgression with less desirable species, such as *O. mossambicus* with several Asian countries experiencing decreased growth in various farmed Nile tilapia strains^4,5^. A similar situation could be affecting productivity in Tanzania, whereby, wild Nile tilapia *O. niloticus* has recently been shown by Bradbeer et al.^6^ and Shechonge et al.^7^ to interbreed with *O. urolepis* and *O. jipe*.

The key factor considered in production of farmed fish is growth performance where a successful development for genetically improving farmed species represent a landmark by which fish production performance in ponds and other culture systems can be sustainably increased^8^. The joint research project called the “Genetic Improvement of Farmed Tilapias” (GIFT) for instance, has played a major role in substantially boosting tilapia production in Asia and the Pacific region^3,5,9^. The project was implemented by ICLARM (currently WorldFish) with the main goal of producing a high performing farmed tilapia strain to a wider range of environments^5^. The GIFT program was based on selective breeding practices that started almost 30 years ago. Since then, there is evidence of sustained gains of 10–15% per generation over more than six generations^10^. The current GIFT strain grows 100% faster than the founder stock^11^ and is considered to be the most popular and favored tilapia strain globally.

Similar selective breeding approaches have been successfully applied to improve various strains of Nile tilapia and other fish species^12–15^. The same technology has also been disseminated to Africa in Egypt and Ghana with a significant increase on Nile tilapia *O. niloticus* production^2,16^. The genetically improved strains of Nile tilapia (*O. niloticus)* developed from all these programs are now reported to be cultivated globally in a wide range of farm environments ^2,3,5^. However, in the presence of genotype by environment interaction (G × E), a strain that has been genetically improved through selection in a favorable environment may not be necessarily good on less favorable conditions^17^.

On the other hand, there are some Asian local tilapia stocks with no genetic improvement but that are widely farmed with an excellent reputation and well-proven track record among aqua-farmers. These include; the red tilapia which have mainly originated through the interspecific cross of *O. mossambicus and O. niloticus*^18,19^ and Chitralada strain from small founder population of *O. niloticus*^3^. Furthermore, a number of farmed tilapia strains have been subjected to hormonal sex reversal, interspecific hybridization (cross-breeding) and genetically male tilapia (GMT) or YY super male technologies. The latter were meant to help avoid problems related to early sexual maturation, unwanted reproduction, and overpopulation in a fish pond^20,21^. Some of these technologies have potential effects on phenotypic traits and environmental fitness of the animals. For example, most of tilapia hybrids yield all-male offspring with increased salt and cold tolerance^22,23^, while heterosis effect on growth performance varied considerably among species and environments^24–26^.

Following the widespread dissemination of the GIFT strain and the adoption of the relevant technology, several studies have investigated the farming potential of GIFT or GIFT-derived strains compared to other improved tilapia strains and local species^27–30^. On the contrary, some selected strains like FaST outperformed GIFT^31^, while, red tilapia and Genomar Supreme Tilapia showed similar performance when cultured at a different temperature range^32^. In addition, in some cases the GIFT strain appeared inferior to local species^3^. Moreover, evidence for genotype by environment interaction has been observed in previous studies^33^. Furthermore, there is evidence of reciprocal introgression between GIFT-derived and Chitralada-derived stocks^34^. Subsequently, some GIFT reference, GIFT-derived and Chitralada-derived populations are facing poor broodstock management and introgression with other feral species e.g. *O. mossambicus* and *O. aureus*^3,34^. Surprisingly, limited research has been performed to assess farming potentials of GIFT and GIFT-derived strains in African environments^16,35,36^, despite the fact that the founder stocks have exclusively originated from the continent^3,5^. For sustainable and profitable aquaculture, continuous evaluation of both GIFT and local breeds should be prioritized.

Potential ecological impacts of tilapia species introductions are broad, exemplified by Nile tilapia (*O. niloticus*), regardless their donation to global aquaculture. The species has proven to interbreed with other native species outside their ecological range^6,7^, caused a decrease in genetic diversity and polluted native endemic populations^37^. Other ecological impacts related to introduced species are competition (food and spawning ground), predation (eggs and larvae of other fish) and habitat alteration^37^. In aquaculture practices, however, careful decision and management on introducing and culture of tilapia species were recommended to reduce the adverse impacts associated to introductions. Canonico et at.^37^ proposed farming of tilapia in contained ponds with no access to natural waters, while Lind et al.^38^ suggested species-specific aquaculture ‘zones’ for tilapia aquaculture.

In Tanzania, aquaculture production (largely due to farmed tilapia) has shown a remarkable growth rate, with an output raising from 3613 tonnes in 2015 to 18,081.6 tonnes in 2018^39,40^ Fast growth, tolerance to environmental fluctuations and easily manageable behavior, make tilapia the fish species with the highest production volume increase in Tanzania with the farming industry based on both local and introduced strains^41,42^. Introduced Nile tilapia strains were imported to increase tilapia farm outputs. Most of the strains were imported by private hatcheries from Asia (Chitralada, BIG NIN and GIFT), Europe (Silver YY) and Uganda, East Africa referred to as “Ruvu Farm” strain (Local breed^41^). However, none of the above hatcheries traces pedigree recordings, less is known about growth performance of their producing fingerlings^43^.

The objectives of this study were (i) to evaluate growth performance of five introduced strains of Nile tilapia (*O. niloticus*) in fresh- and brackish-water environments and (ii) to determine whether there was a presence of genotype by environment interaction (G × E) between both locations. Evidence shows that the climatic conditions of the tested environments do not really vary^44,45^. The study hypothesized that there will be comparable growth performance among the five tested strains regardless of locations. It was also hypothesized that there will be no significant difference in ranking on growth performance among tested strains between environments. Our findings reveal the aquaculture potential of each strain tested in different environments. These findings are expected to inform planned tilapia breeding programs in Tanzania and elsewhere, particularly in sub-Saharan African countries challenged with freshwater shortage and saltwater intrusion in coastal areas. Furthermore, our results provide awareness for a successfully reared strain(s) both in fresh- and brackish-water environments.

## Materials and methods

### Experimental sites

The trials were conducted at two stations: (i) the Institute of Marine Sciences Mariculture Centre (IMS-MC) at Pangani district in Tanga, situated at approximately 05° 25′ 58″ S, 038° 57′ 45″ E in the southern bank of Pangani River on the Pemba Channel of the Indian ocean. The recorded water salinity in this site was 2 units and characterized by annual rainfall above 1000 mm and temperature varying between 25 and 33 °C. (ii) the School of Aquatic Science and Fisheries Technology, University of Dar es Salaam at Kunduchi in Kinondoni district in Dar es Salaam, Tanzania, situated at approximately 6° 40′ 0″ S, 39° 13′ 0″ E along the coastline of Indian Ocean. Annual temperature and rainfall in Dar es Salaam varies little throughout the year with average of 25 °C and 1089 mm. Overall, small variations of climatic conditions were observed between the two stations due to the influence of the Indian Ocean. The test environments chosen for the needs of this study correspond to water types commonly encountered in tilapia farming in Tanzania: (i) brackish water from estuaries, coastal aquifers or rivers encroached by coastal waters and (ii) freshwater from deep wells, dams, river, municipal water and lakes.

### Hatchery seed production

Tilapia hatcheries had similar practices for seed production, whereby, four to eight hapas were arranged in one pond (150–700 m^2^). The brood fishes (100–300 individuals) were pooled in one hapa (27–60 m^2^) following a common stocking ratio of one male to three females. Then, eggs were collected from the fish mouth and incubated in special hatching jars. Incubation of eggs was mostly performed in indoor facilities with recorded annual average temperature ranging between 23.3 and 25 °C.

### Experimental fish

#### Collection of fish and transport techniques

In this study, fry of the introduced Nile tilapia strains (referred to as: BIG NIN, GIFT, Chitralada, “Ruvu Farm” and Silver-YY; *n* = 400 per strain) were collected immediately after hatching from four different hatcheries namely; Mpanju Fish Farm, Rift Valley, Ruvu Farm and Shazain/JAN’s Farm (Table 1) from June to July, 2019. All fry were first transported to Pangani Centre and, after hormonal treatment; half of the fish from each strain were transported to Kunduchi Centre. Transportation was done during daytime using a car. During transport, fry were packed in plastic bags containing approximately five liters of water filled with pure oxygen gas and placed in plastic containers measuring 20 L volume each. To reduce fish stress and ensure survival rate during transport, the automotive air conditioning system was used to cool or warm to desired environment in the car.

**Table 1:** Sampling locations in Tanzania and country of origin of Nile tilapia strains used in the trials.

Strain	Hatchery	District	Region	Origin
BIG NIN	Mpanju Fish Farm	Misungwi	*Mwanza*	Nam Sai Farm-Thailand
GIFT	Mpanju Fish Farm	Misungwi	*Mwanza*	Nam Sai Farm-Thailand
Silver-YY	Rift Valley	Kinondoni	*Dar es Salaam*	Til-Aqua Inter-Netherland
“Ruvu-Farm”	Ruvu Farm	Bagamoyo	*Pwani*	AA Fisheries and Aqua Cons LTD-Uganda
Chitralada	Shazain/JAN’s Farm	Mkuranga	*Pwani*	Asian Institute of Technology-Thailand

#### Acclimatization to brackish water

Since all fish were collected from freshwater environments, fry from each strain were gradually acclimatized for thirty minutes to brackish water (salinity = 2) on their arrival at Pangani. Acclimatization was done by gently pouring the brackish water with intervals into plastic bags which contained the fish and freshwater. All dead individuals (BIG NIN = 30; GIFT = 120; Silver YY = 0; “Ruvu Farm” = 3; Chitralada = 2) were removed and counted to estimate the number of remaining experimental fish. Fry from each strain were separated into four groups and stocked in 80-L labeled plastic basins at a stocking density of 1 fry L^−1^ until they were subjected to hormonal sex reversal treatment.

### Preparation of hormonal treated feed

One kilogram of hormonal treated feed was prepared at the National Fish Quality Control Laboratory (NFQCLAB), Mwanza, Tanzania using untreated starter feed measuring 0.8 mm from Koudijs Animal Nutrition-Tanzania Ltd. Koudijs’ diet contained 400 g/kg crude protein, 65 g/kg crude fat, 40 g/kg crude fiber, 110 g/kg moisture, 140 g/kg ash and 245 g/kg nitrogen-free extract. Sixty milligrams of alpha methyltestosterone hormone (17a-MT; Sigma Aldrich, China) was dissolved in one liter of 95% ethanol and placed on laboratory hot plate magnetic stirrer (FAITHFUL, model SH-2, Huanghua, China) to make a homogenous standard solution. The solution was evenly sprayed over one kilogram of the Koudijs’ diet and mixed by hand several times to ensure homogeneity. The treated feed was spread onto four rectangular stainless-steel plates (lab trays) overnight at 25 °C to allow a total evaporation of ethanol. The dried hormonal treated feed was then sealed in special container and stored at 4 °C in a refrigerator until use.

### Production of mono-sex populations

All fish in 80-L labeled plastic basins except silver YY groups (males by YY super male technology) were subjected to hormonal sex reversal treatment. Hatchlings were fed with the hormonal treated diet to satiation three times daily between 0900–0930, 1400–1430 and 1700–1730 h for 28 days. Silver YY groups were fed the same Koudijs feed with no hormone addition. Chitralada fry started to receive a hormonal dose from 15th June to 13th July, 2019 followed by “Ruvu Farm”, BIG NIN and GIFT from 23rd July to 21st August, 2019. Routine water exchange of 100% and siphoning was performed on a daily basis to remove uneaten food, fish wastes and other debris. Likewise, to ensure regular supply of air throughout the trial, water aeration was done using aquarium bubblers connected directly to air compressor electric motor (Single phase, volts 220 V–50 Hz/60 Hz, model YL90L-2, Zhejiang, China).

### Rearing of fingerlings

The nursery phase started soon after hormonal treatment where half of the fish from each strain (BIG NIN = 181; GIFT = 101; Silver = 104; “Ruvu Farm” = 150; Chitralada = 175) were transferred to Kunduchi Centre for rearing in a freshwater environment. All fingerlings were acclimatized using same technique that was initially used at Pangani as previously described in this article. All fish at both stations were randomly assigned to labelled hapa nets measuring 1 m × 2 m × 1.5 m arranged in liner and concrete ponds measuring 20 m × 20 m each at Pangani and Kunduchi, respectively. The stocking density for each strain was 40 fry/m^2^. All fry from each strain were captured with a fingerling scoop net of 1 mm mesh size and placed to a small plastic container on top of a calibrated sensitive weighing balance to nearest 0.01 g (Boeco, model 43, Hamburg, Germany). Chitralada and Silver YY fry (average weight of 1.40 g) were firstly transferred to hapas on 21st July followed by “Ruvu Farm” (average weight of 0.56 g), BIG NIN and GIFT (0.004 g) on 24th August 2019. Fish were fed to satiation three times a day at around 0900, 1300 and 1700. Regular cleaning of hapas was done to remove uneaten food, fish waste and other debris that could accumulate on net bottom and on the walls. There was a biweekly pond water exchange of 20% to ensure sufficient dissolved oxygen supply and reducing levels of turbidity due to uneaten feed. The time duration of each strain in a nursery phase before grow-out experiment is provided in Table 2.

**Table 2:** Stocking, harvesting and age effect mean weights (± s.e) of Nile tilapia strains reared at Kunduchi and Pangani experimental sites.

Location	Strain	*N*	At stocking for grow-out		At harvesting		Mean weight (g; age effects)
			SW	Mean weight (g)	FSW	Mean weight (g)	
Kunduchi	BIG NIN	168	14	81 ± 2.19	22	285 ± 4.92	195 ± 4.01^a^
	GIFT	61	14	92 ± 3.64	22	298 ± 8.17	208 ± 5.93^a^
	Silver YY	64	19	89 ± 3.55	27	201 ± 8.24	101 ± 5.46^b^
	“Ruvu Farm”	107	15	57 ± 2.75	23	188 ± 6.20	138 ± 4.73^c^
	Chitralada	71	20	140 ± 3.37	28	286 ± 8.40	157 ± 6.13^c^
Pangani	BIG NIN	82	14	122 ± 3.14	22	333 ± 7.43	238 ± 4.87^d^
	GIFT	54	14	116 ± 3.87	22	370 ± 9.10	251 ± 6.10^d^
	Silver YY	69	19	101 ± 3.42	27	241 ± 7.59	145 ± 5.52^e^
	“Ruvu Farm”	79	15	46 ± 3.20	23	234 ± 7.32	181 ± 5.07f.
	Chitralada	41	20	127 ± 4.44	28	350 ± 9.63	200 ± 6.34f.

Superscripts shown at each mean weight (age effect) indicate the various levels of significant difference (p < 0.0001) by the F- test. *n*-Number of samples, SW-Survived weeks from fry collection to stocking, FSW-Final survived weeks from fry collection to harvest.

### Tagging and grow-out stocking

When all fish attained a weight exceeding 20 g both at Pangani and Kunduchi, individuals from each strain were tagged in the abdomen cavity with Passive Integrated Transponder (PIT-tag; 2 × 12 mm) using a syringe implanter. A mixture of 0.2 ml of clove oil per liter of water was used to anesthetize fish before tagging. A PIT tag reader (R5-pro; Europe) was used to scan the fish. Fish were then stocked into seven different grow-out hapas measuring 12 m × 8.5 m × 2 m each aligned into ponds measuring 20 m × 20 m. Two concrete ponds with two hapas each at Kunduchi and two lined ponds at Pangani one with two hapas and another with one hapa were used. All fish were stocked in a completely randomized block design, whereby, every strain had almost equal representatives in each single hapa at both stations. The fish were stocked at 5 fish/m^2^. Prior to stocking fish for growth performance, manual sexing was done to remove all females that did not respond to hormonal sex reversal treatment. The total number of fish that were subjected to growth-out experiment were 471 animals at Kunduchi (BIG NIN = 168; GIFT = 61; Silver YY = 64; “Ruvu Farm” = 107; Chitralada = 71) and 325 animals at Pangani (BIG NIN = 82; GIFT = 54; Silver YY = 69; “Ruvu Farm” = 79; Chitralada = 41; Tables 2, 4). Experimental fish were fed formulated pellet feed that contained 350 g/kg crude protein and fed until satiation twice daily between 0900 and 1000 and 1500 and 1600 h. The grow-out period lasted for about eight weeks at both stations. Unfortunately, a week before finalizing the experiment, two hapas of the same pond at Pangani overturned due to severe winds and mixed the fish together, therefore creating difficulties to include hapa effects in the analysis.

### Morphometric measurements

Body weight and length were measured using a sensitive weighing balance (Boeco, model 43, Hamburg, Germany) and a measuring ruler, respectively. Initial fish body weight (g), depth (cm), girth (cm), total length (cm) and standard length (cm) were recorded before stocking fish into grow-out hapas. Likewise, final parameters were determined at harvest (Tables 2, 4). Tag number and parameters of all fish that died were also recorded. Numbers of fishes from each strain at harvest were recorded and used to calculate percentage survival (Table 2). Temperature, DO and pH were monitored and recorded throughout the experiment using a portable oxygen meter (Hanna, model HI 98193, Hanna Instruments Inc, Woonsocket, Rhode Island, USA).

### Survival rate and sex ratio

An average percentage of fish confirmed to be males from each strain at both stations and survival rate were calculated as follows:

$${\text{\% of sex reversed male fish}} = \frac{{\text{Sex reversed male fish at stocking }}}{{\text{Total number hormonal treated fish at stocking}}} \times { }100,$$

$${\text{Survival rate }}\left( {\text{\% }} \right) = \frac{{\text{Final number of fish}}}{{\text{Initial number of fish}}} \times { }100.$$

### Statistical analysis

#### Correlation for morphometric traits

The phenotypic correlations between the morphometric traits were computed by Pearson’s linear correlation coefficient (*r*) using the ggpubr R package version 0.2.4^46^. Shapiro–Wilk test^47^ using R function: shapiro test (R) was used as a preliminary test to check for the normality of data, and *r* was interpreted as previously described by Evans^48^ and Cohen^49^. Pearson’s formula used to measure a linear dependence between two dependent variables (*x* and *y*) at confidence interval of 95% was:

$$r = \frac{{\sum \left( {x - m_{x} } \right)\left( {y - m_{y} } \right)}}{{\sqrt {\sum (x - m_{x} )^{2} \sum (y - m_{y} )^{2} } }},$$

where, *x* and *y *are two vectors of length n (sample size), *m*_*x*_ and *m*_*y*_ are the means of *x* and *y* variables, respectively.

#### General estimates of variances

Individual strain performance was determined as:

$$G_{w\lg } = F_{w\lg } - I_{w\lg } ,$$

where *G*_*wlg*_ is the mean gain body weight, length or girth; *F*_*wlg*_ is the mean final body weight, length or girth; *I*_*wlg*_ is the mean initial body weight, length or girth.

Analysis of variance was implemented using R v.3.5.3 (R Core Development Team, 2018). The ANOVA type III for unbalanced data was performed using the R package Car version 3.0-7^50^ to evaluate for potential interactions (location and strains) and estimate differences between strains for the dependent variables (body weight, and girth). The mean of the independent variables was compared by the package emmeans version 1.4.5^51^ using Tukey method and declared significant when p < 0.05. Upon significant interaction between independent variables mean comparison was done by nesting variables against one another in order to avoid misleading results according to R. To correct for the differences between age of strains and final weight at harvest, age effects was included in linear model as a confounding factor during the statistical analysis. The linear model used for each trait was:

$$y_{ijk} = \mu + \alpha_{i} + \, \beta_{j} + (\alpha \beta )_{ij} + z_{ijk} + \, e_{ijk} ,$$

where, μ is the general mean, *y*_*ijkl*_ is the observed weight gain (g), length (cm), girth (cm), *α* is the strain effect, *β* is the rearing environment effect, *αβ* is an interaction effect of strain and environment, *z*_*ijk*_ is the age effect, *e* is the residual effect.

### Ethical statement

The study protocol was approved on 14 August, 2018 by the Institute’s Postgraduate Studies Committee (IPSC; No. 2016-07-00166) of University of Dar es Salaam (UDSM) in compliance with the Tanzania Fisheries Act (2003) and the Wildlife Conservation Act, 1974. We confirm that the study was undertaken with all procedures which minimize pain, suffering and improve animal welfare. Permit to collect and transport all fish strains from hatcheries to experimental stations was issued by Department of Aquaculture Development, Ministry of Livestock and Fisheries and other local authorities for complying with the requirement of Fisheries Regulations, 2009 (G.N. No. 308 of 2009).

### Reporting guidelines

We used in **vivo** experiment. The study is reported in accordance to the ARRIVE guidelines (Animal Research: Reporting of In Vivo Experiments).

### Collection-transport permit

Permit to collect and transport fish (Nile tilapia strains) from hatcheries to experimental stations was issued by Department of Aquaculture Development, Ministry of Livestock and Fisheries, to ensure compliance with all conservation and fishery management measures provided in Tanzania Fisheries Act of 2003.

## Results

### Fish mortality and pond conditions

Number of deaths recorded during hormonal sex reversal trial were 8, 77, 96, 48 and 192 for BIG NIN, GIFT, “Ruvu Farm”, Chitralada and Silver YY, respectively. Overall percentage of fish mortality of all tested strains after the grow-out period of eight weeks from both locations of Pangani and Kunduchi were relatively low at 10% (Table 3). Mortality does not appear to be location specific and were more or less similar in both locations. The Chitralada strain recorded the highest mortality (23%) at Pangani, followed by BIG NIN (17%) at Kunduchi. Other strains registered mortalities that ranged from 4 to 15% at both locations (Table 3). No tag loss was observed to any individual fish at harvest.

**Table 3:** Mean body weight gain, standard length, girth and mortality percentages of grow-out in hapas of five Nile tilapia strains reared at Kunduchi and Pangani.

Location	Strain	N	Weeks	Weight gain (g)	DWG (g)	Standard length (cm)	Girth (cm)	Mort (%)
Kunduchi	BIG NIN	168	8	204 ± 4.45^a,i^	3.64 ± 0.38^a,i^	6.11 ± 0.16^a,g^	6.74 ± 0.13^a^	17
	GIFT	61	8	206 ± 7.39^a^	3.67 ± 0.40^a^	5.94 ± 0.27^a^	6.37 ± 0.21^a,d^	15
	Silver YY	64	8	108 ± 7.46^b^	1.92 ± 0.40^b^	3.83 ± 0.27^b^	3.74 ± 0.21^b,e^	4
	“Ruvu Farm”	107	8	131 ± 5.61^b,c^	2.34 ± 0.39^b,c^	5.44 ± 0.20^a^	5.34 ± 0.16^c^	10
	Chitralada	71	8	140 ± 7.59^c^	2.50 ± 0.40^c^	3.87 ± 0.27^b^	4.04 ± 0.22^b^	0
	Over-all	471	–	166 ± 51.36	2.97 ± 0.92	5.34 ± 1.42	5.62 ± 1.54	10
Pangani	BIG NIN	82	8	218 ± 6.77^d,i^	3.84 ± 0.40^d,i^	6.24 ± 0.24^d,g^	5.81 ± 0.19^d^	7
	GIFT	54	8	254 ± 8.23^e^	4.54 ± 0.40^e^	7.02 ± 0.30^d,f^	6.60 ± 0.24^d^	7
	Silver YY	69	8	139 ± 6.87^f^	2.48 ± 0.40^f^	4.66 ± 0.25^e^	4.12 ± 0.20^e^	13
	“Ruvu Farm”	79	8	191 ± 6.67^g^	3.36 ± 0.39^g^	7.80 ± 0.24^f^	7.44 ± 0.19^f^	7
	Chitralada	41	8	223 ± 8.71^d,e^	3.97 ± 0.41^d,e^	6.16 ± 0.31^d^	5.73 ± 0.25^g^	23
	Over-all	325	–	200 ± 60.38	3.56 ± 1.09	6.37 ± 1.64	5.94 ± 1.66	10

Means of different strains within a location and the same strain between locations followed by different superscripts in the same column significantly differ (p < 0.05) by the F-test. *N* = number of samples, S = % *survival rate.*

During the grow-out experiment, pond water temperature ranged from 29.2 to 37.6 °C and 27.7 to 36.5 °C at Pangani and Kunduchi, respectively. Likewise, dissolved oxygen (DO) ranged from 2.5 to 10.7 mg/L and 2.6 to 7.9 mg/L in the morning and from 1.3 to 14.5 mg/L and 3.5 to 9.5 mg/L in the afternoon at Pangani and Kunduchi, respectively.

### Percentages males

An average percentage of fish confirmed to be males from each strain at both locations were 84%, 74%, 100%, 82% and 66% for BIG NIN, GIFT, Silver YY, “Ruvu Farm” and Chitralada, respectively.

### Growth

**Figure 1:**
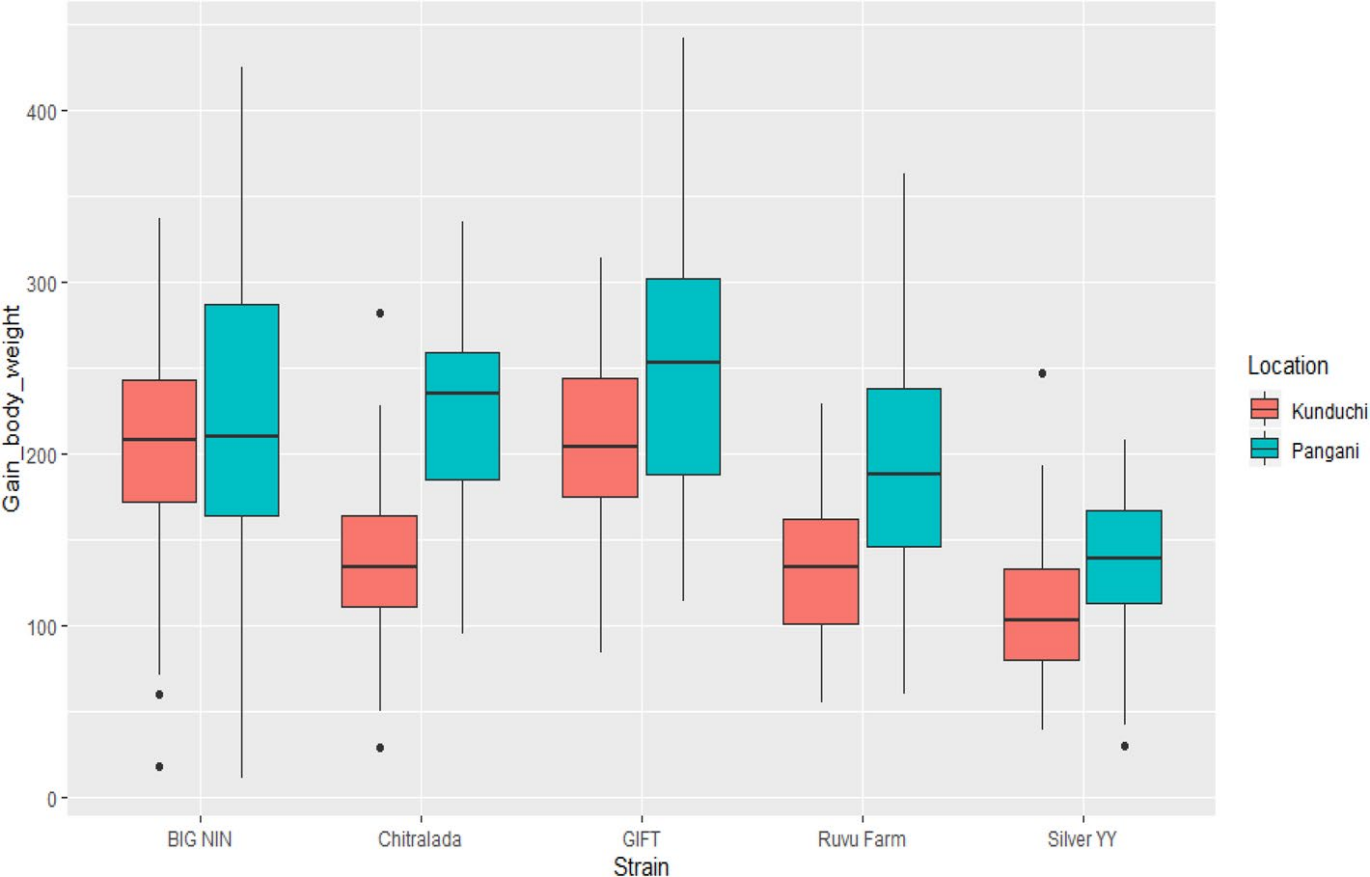
Box plots representing mean body weight gain of Nile tilapia strains reared at Kunduchi and Pangani.

Results for mean final body weight indicated that the GIFT strain attained the highest weight gain at both locations, followed by Chitralada, BIG NIN, Sliver YY and “Ruvu Farm” (Table 2). However, substantial differences for body mean weight gain were seen between strains in the same location and within strain across the tested environments. The results from body weight gain indicated that GIFT and BIG NIN (GIFT-derived) strains had significantly higher weight gain (p < 0.001) compared to Silver YY, “Ruvu Farm” and Chitralada strains at Kunduchi (Table 3; Fig. 1). Overall, the GIFT strain showed 1%, 47%, 57% and 90% faster growth rate than BIG NIN, Chitralada, “Ruvu Farm” and Silver YY strains, respectively, at Kunduchi. Likewise, the GIFT strain grew 14%, 17%, 33%, and 83% faster than Chitralada, BIG NIN, “Ruvu Farm” and Silver YY at Pangani, respectively (Table 3; Fig. 1). However, no significant differences were observed in mean weight gain between GIFT and Chitralada strains as well as between BIG NIN and Chitralada strains at Pangani. Likewise, the “Ruvu Farm” strain showed no significant differences in body weight gain when compared to the Silver YY and Chitralada at Kunduchi, Chitralada showed significantly better performance (p < 0.05) over the Silver YY and “Ruvu Farm” strains at Pangani. Moreover, the weight gain in Silver YY strain was significantly lower (p < 0.05) compared to all tested strains from both locations except to “Ruvu Farm” and Chitralada at Kunduchi.

**Table 4:** Correlation coefficient among the morphometric traits of five Nile tilapia strains reared at Kunduchi and Pangani.

		Morphometric traits		Correlation coefficient (*r*)		
		Trait 1	Trait 2	Kunduchi	Pangani	Overall
Strain	BIG NIN	BWG	SLG	0.43	0.81	0.55
		BWG	BGG	0.78	0.88	0.77
		SLG	BGG	0.39	0.82	0.50
	GIFT	BWG	SLG	0.67	0.57	0.64
		BWG	BGG	0.74	0.88	0.78
		SLG	BGG	0.84	0.57	0.67
	Silver YY	BWG	SLG	0.79	0.82	0.83
		BWG	BGG	0.85	0.84	0.83
		SLG	BGG	0.86	0.86	0.85
	“Ruvu Farm”	BWG	SLG	0.77	0.70	0.81
		BWG	BGG	0.79	0.85	0.88
		SLG	BGG	0.77	0.82	0.87
	Chitralada	BWG	SLG	0.88	0.89	0.89
		BWG	BGG	0.92	0.89	0.89
		SLG	BGG	0.93	0.92	0.92

Morphometric traits: Body weight gain (BWG); Standard length gain (SLG); Body girth gain (BGG).

The results for body weight gain across the tested environments showed that, almost all tested strain groups such as GIFT, Silver YY, “Ruvu Farm” and Chitralada at Pangani performed significantly better (p < 0.05) than their respective counterparts at Kunduchi except for the BIG NIN strain (Table 3; Fig. 1). The results indicated that, the GIFT, BIG NIN, Silver YY, “Ruvu Farm” and Chitralada strain grew 23%, 7%, 29%, and 59% faster than their respective counterparts at Kunduchi, respectively (Table 3; Fig. 1).

**Figure 2:**
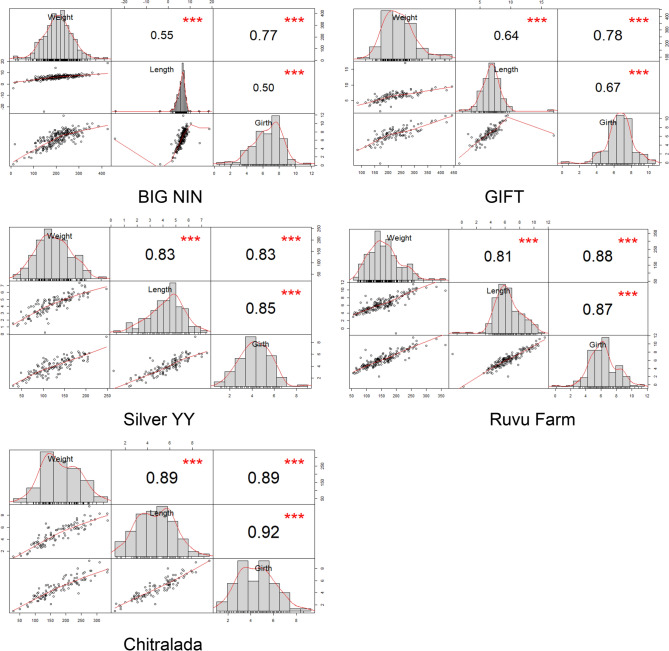
Correlation coefficient representing gained body weight, standard length and girth of Nile tilapia strains reared at Kunduchi and Pangani.

### Age effect

The results of the age effect displaying adjusted final mean weights at harvest for all tested strains (Table 2). The results showed that GIFT and BIG NIN strains attained significantly higher average mean final weights than Chitralada “Ruvu Farm” and Silver YY (p < 0.0001) both at Kunduchi and Pangani (Table 2). Similarly, “Ruvu Farm” and Chitralada were found to have attained significantly higher final mean weight than Silver YY (p < 0.0001) in both tested environments.

### Morphometric traits

The BIG NIN, GIFT, silver YY and Chitralada from both Kunduchi and Pangani experimental stations showed a close similarity for standard length gain (SLG) and body girth gain (BGG). The “Ruvu Farm” strain represented morphometric values disproportionate to its mean body weight gain (BWG) compared to other strains (Table 3). The GIFT, BIG NIN and “Ruvu Farm” strains showed no significant difference (*p* > 0.05) for SLG at Kunduchi, similar to Chitralada and Silver YY strains. The “Ruvu Farm” strain showed significantly higher SLG and BGG (*p* < 0.05) compared to all tested trains at Pangani, except for GIFT with SLG. No significant difference was observed from GIFT and BIG NIN strains for SLG and BGG across the tested environments. In addition, the GIFT and BIG NIN strains showed significantly higher BGG (*p* < 0.05) than other tested strains at Kunduchi. Significant lowest SLG and BGG among all tested strains and environments were observed from Silver YY, with exception to Chitralada’s BGG at Kunduchi.

### Correlations amongst recorded traits

The correlations for BWG, SLG and BGG for the BIG NIN, GIFT, Silver YY, “Ruvu Farm” and Chitralada depicts weak and strong positive correlation coefficients (*r*) among all morphometric traits both at Kunduchi and Pangani (Table 4, Fig. 2). The Chitralada, Silver YY and “Ruvu Farm” strains showed the highest positive correlation coefficient values for all traits at both locations. Weak and moderate correlation values were seen in BIG NIN and GIFT strains at Kunduchi and Pangani, respectively.

Overall results indicated that, the Chitralada strain had the highest correlations amongst the recorded traits (BWG/BGG = 0.89; SLG/BGG = 0.92) followed by “Ruvu Farm” (BWG/BGG = 0.88; SLG/BGG = 0.87). The lowest correlations were observed in BIG NIN (BWG/SLG = 0.55; BWG/GBG = 0.77; SLG/BGG = 0.50). The BIG NIN, GIFT and “Ruvu Farm” strains showed highest BWG/BGG values of 0.77, 0.78 and 0.88 in comparison to other correlations, respectively. In contrast to Silver YY and Chitralada strains which showed highest values of 0.85 and 0.92 in SLG/BGG, respectively. Generally, the correlations values for BWG/SLG were lowest to all tested strains except for GIFT strain. Furthermore, a slight difference in correlations values for all traits were seen between the Silver YY and “Ruvu Farm” strains (0.06), followed by Chitralada and “Ruvu Farm” strains (0.11).

## Discussion

The present study was conducted to assess the growth performance of five different strains of Nile tilapia (*Oreochromis niloticus*) introduced to Tanzania reared in fresh- and brackish-waters. Our findings negate the hypothesis set earlier that there will be no significant difference in growth performance among the five strains, regardless of the locations. Our results across the tested environments, showed clear differences between locations and lines. On the contrary, the genotype–environment interaction (G × E) among strains between environments supported the hypothesis predicted in this study. The results from present study showed that no strong genotype–environment interaction (G × E) was observed since the ranking between lines appeared to be consistent across the different locations with the exception of the BIG NIN and Chitralada strains.

The obtained results indicate that GIFT ranked first at both locations, followed by BIG NIN and Chitralada at Kunduchi and Pangani, respectively, whereby “Ruvu Farm” and Silver YY ranked fourth and fifth at both stations. Similar findings have been reported from other studies for GIFT and BIG NIN (GIFT-derived; http://tilapiathai.com/project/nile-tilapia/), given that GIFT is an improved strain with proven growth potential in a wide range of environments^3,5,52^. The observed performance range of 14–90% from the present study, is greater than those reported from a series of on-farm trials (18–58%) on superior performance of the GIFT strain compared to the non-GIFT strains^52^. The study was conducted by ICLARM and its partner organizations in diverse agro-ecological zones in five Asian countries such as Bangladesh, China, Philippines, Thailand and Vietnam. The lower range reported by the latter study was associated with cold weather in some Asian countries especially China, since GIFT strain has lower cold tolerance than other farmed Nile tilapia strains^5^. This is also supported by Sifa et al.^53^, where poor cold tolerance of the GIFT strain was observed when compared to Sudanese and Egyptian ones. Thus, higher performance of GIFT in this study could be associated with the higher temperature range from 27.7 to 37.6 °C recorded throughout the experiment, which appears to be the most suitable for GIFT performance^32^. However, the higher recorded temperature above 32 °C in this study is beyond the optimum range for some tilapia species. Such high temperature could negatively affect the growth of fish in the experiment^54^. Therefore, the GIFT and GIFT-derived strains could be the best choice to grow in high temperature environment range from of  27.7 to 37.6 °C.

The present results further indicate that Chitralada strain attained higher performance in brackish water than “Ruvu Farm” and Silver YY. Moreover, Chitralada strain’s performance was similar with those of either GIFT or BIG NIN strains. The findings of the current study in brackish water corresponds with those by Nandlal et al.^55^ who observed a minor growth performance of the GIFT strain compared to Chitralada strain in freshwater environment. Previous study had also proven the growth potential of GIFT strain over descendants of pure Chitralada strain in freshwater environment^56^. In our study, the higher initial weight and age of the Chitralada strain at stocking from both Kunduchi and Pangani are likely to have contributed to the higher body weight gain encountered in this strain. Furthermore, the close growth range seen at Pangani from GIFT and BIG NIN with other strains such as Chitralada, “Ruvu Farm” and Silver YY at Pangani could be associated with earlier higher weight achieved by GIFT and BIG NIN and resulted to sluggish growth. This is a basic principle for the animal growth rate, as the growth rate is greatest in earlier ages and decreases with increase in age and size^32,57^.

In this study we expected to find higher growth performance in Silver YY strain, as the Til-Aqua International in Netherlands developed the tested strain from various lines including GIFT’s gene pool (https://www.til-aqua.com/yy-technology/), under appropriate hatchery management^58^. However, the observed Silver YY strain’s growth performance in this study was significantly lower compared to all tested strains across the tested environments except to “Ruvu Farm” strain at Kunduchi. The lower Silver YY strain’s growth performance reported from the current study probably resulted from the effects of aggressive interactions, which is common to fin fish^59^. This, however, may not be the only reason, as fish groups in this study were formed by heterogeneous sized fish which underlines the social position as well as defining quickly the hierarchy without high levels of aggression^60,61^. Therefore, further investigation is required to clear this doubt, because previous studies show heterosis effect on growth performance of farmed tilapia vary considerably among species and environments^24,26,62,63^.

Interestingly, the overall weight gain and daily weight gain from BIG NIN, GIFT, Silver YY, “Ruvu Farm” and Chitralada in this study are higher than the equivalent strains as reported elsewhere^64–67^. The achieved body weight gain in the present study could be probably attributed by a medium optimal stocking density of 5 fish/m^2^ (~ 200 fish/dec or 50,000 fish/ha) used in the study. The stocking density used in our study is almost comparable to that recommended in Shoko et al.^68^. Although it is not supported by the findings from Hasan et al.^69^ and Rahman et al.^70^ who recommended a stocking density of 150 and 125 fish/dec as an optimum for GIFT and mono-sex tilapia population respectively. Interestingly, the same range of daily weight gain for the GIFT strain was observed in the recirculating aquaculture system (RAS) using tanks^71,72^.

When calculating the morphometric traits in the present study, GIFT and BIG NIN demonstrated significantly higher mean SLG and BGG than all tested strains across the tested environments, except for “Ruvu Farm” strain at Pangani. The higher values showed by GIFT and BIG NIN (GIFT-derived) in this study, could be connected to the estimates of heritability of medium to high magnitude for GIFT strain presented by Reis Neto et al*.*^73^. Interestingly, the results from Pangani showed that “Ruvu Farm” strain to have significantly higher SLG and BGG than all tested strains, except for the GIFT’s SLG. Generally, individuals from “Ruvu Farm” were more elongated and had more size from anterior insertions of dorsal fin to anterior origin of pelvic fin as well as big heads than other strains. The GIFT and BIG NIN strains showed deep but laterally compressed bodies and smaller head compared to other strains. Chitralada and Silver YY somehow resembled each other with elongated bodies and medium head size. The linear body measurements which include length and girth are not short-term changes, as they are used to describe an animal more completely than conventional methods of weighing and grading^74,75^. The morphometric traits evaluated in this study have a good potential that might be possible for selection when breeding. However, it is common phenomenon for farmed tilapia to display differences in body shape^76^.

Generally, our results showed a clear difference in performance for body weight, length and girth between the two locations for almost all strains (BIG NIN, GIFT, Silver YY, “Ruvu Farm” and Chitralada). Overall higher growth was observed at Pangani except for the BIG NIN strain. At both locations, water conditions such as temperature and dissolved oxygen (DO) did not vary appreciably between locations throughout the experiment. All fingerlings transported to Kunduchi had sufficient time to recover from transport stress before stocking as previously described in this article. The best performance of the fish cultivated in brackish water (lower salinity) at Pangani could be related to the lower energy cost for the ionic regulation, as reserving energy can be directed towards growth^77^. Findings from the present study agrees with those of Azevedo et al.^78^ who observed higher weight gain and feed intake in Nile tilapia as salinity increased from 0 to 7 g L^−1^.

In the present study, morphometric traits for all studied strains have presented moderate and strong positive correlations, except for BIN NIN at Kunduchi which showed a weak correlation of standard length in relation to body girth. The present correlation values are within the ranges reported in Nile tilapia from previous study 79. Regarding GIFT strain, for example, previous studies^73,79^ reported higher correlation values among the morphometric traits. However, this could not be associated with genetic reduction, as individuals from the same gene pool may express different morphometric due to divergence in the rearing environment^80–83^.

The overall results here suggest that there is a clear difference in growth performance between the five introduced strains (BIG NIN, GIFT, Silver YY, “Ruvu Farm” and Chitralada) both in fresh- and brackish-water environments in Tanzania. However, no strong genotype–environment interaction (G × E) was observed as the ranking among lines remained relatively the same across the tested environments. The observation is in agreement with findings of previous studies which tested the need for breeding specific tilapia strain regarding the environment factors or farming systems^12,84^.

## Conclusion

We provide clear evidence that the growth performance of all five introduced strains of Nile tilapia (*O. niloticus*) imported from Thailand (BIG NIN, GIFT and Chitralada), Uganda (“Ruvu Farm”) and the Netherlands (Silver YY) showed clear differences between both locations and lines. We also found that no strong genotype–environment interaction (G × E) was observed as the rankings between lines were consistent across locations. Therefore, a strategy for the development of specialized strains for freshwater and low salinity brackish water (including salt-intruded) water environments in tilapia breeding program is not necessary. However, a salt tolerant strain of tilapia is needed to utilize the vast potential of sea water in Tanzania and adapt to the areas which have been severely impacted with saltwater intrusion. Furthermore, we observed that the introduced tilapia strains in Tanzania display differences in body shape. As traits of this category are easily identified by consumers, it could be valuable to include it in a selective breeding scheme. Overall, the future tilapia breeding in Tanzania, should consider that *O. niloticus* is the most widely cultivated species in the country. Therefore, breeding for its better performing strains may be prioritized taking full advantage of capacities of both introduced and native tilapia strains present in Tanzania.

## Data Availability

Data generated from this study will be made available upon request.
